# Data Analytics and Applications of the Wearable Sensors in Healthcare: An Overview

**DOI:** 10.3390/s20051379

**Published:** 2020-03-03

**Authors:** Mohy Uddin, Shabbir Syed-Abdul

**Affiliations:** 1 Executive Office, King Abdullah International Medical Research Center, King Saud bin Abdulaziz University for Health Sciences, King Abdulaziz Medical City, Ministry of National Guard—Health Affairs, Riyadh 11426, Saudi Arabia; drmohyuddin@yahoo.com; 2 Graduate Institute of Biomedical Informatics, Taipei Medical University, Taipei 10675, Taiwan

## 1. Introduction

Improving health and lives of people is undoubtedly one of the prime goals of healthcare organizations, policy-makers, and leaders around the world. The need of ageing, disability, long-term care, and palliative care in our current society pose formidable challenges for disease burden and healthcare systems that must be addressed [1]. In order to tackle the leading causes of morbidity and mortality that may result from infections to chronic conditions especially in older adults and ageing population, the accessibility and provision of long-term care and palliative care, when and where needed by them, is crucial. With the continuous challenges and rising demands of the elderly, remote and home-based care, the technological innovations in the fields of digital health and health information and communication technologies, such as mobile health, wearable technologies, telemedicine and personalized medicine have transformed the ways of practice and delivery of healthcare in the recent decades [2]. Wearable technologies have been extensively used in the healthcare sector with multi-purpose applications ranging from patient care to personal health. In clinical and remote care, the applications of wearable devices/sensors, mobile applications, and tracking technologies are of immense importance for the diagnosis, prevention, monitoring, and management of chronic diseases and conditions [3]. The data generated from the wearable devices/sensors are a cornerstone for healthcare data analytics, especially when it is utilized by latest technologies, such as Artificial Intelligence (AI), Machine Learning (ML), Big Data Intelligence, and Internet of Things (IoT) Systems. The literature has many successful examples of utilization of these data in various branches of medicine, such as oncology, radiology, surgery, geriatrics, rheumatology, neurology, hematology, and cardiology. With the regular ongoing updates, the outcomes of data analytics and their applications are already making a huge impact in transforming and revolutionizing the healthcare industry.

In this special issue, we aim to provide new insights on research data analytics and applications of wearable devices/sensors in healthcare by covering wide range of related topics. This issue represents the latest research that spans across 19 countries, 37 institutions and is covered by a total of 28 articles. To make better understanding of the research articles, we have arranged them in an order to show various covered aspects in this field, such as technology integration research, prediction systems, rehabilitation studies, prototype systems, community health studies, detection systems, ergonomics studies, technology acceptance studies, monitoring systems, warning systems, sports studies, clinical systems, feasibility studies, parameters measurement systems, design studies, location based systems, tracking systems, observational studies, risk assessment studies, activity recognition systems, impact measurement systems and systematic review. 

## 2. Summary of Special Issue Papers

In order to provide a basic overview, we will go through and provide brief summary of all the articles of wearable devices/sensors covered in this issue one by one. Bayo-Monton et al. [4] provided an implementation of new portable system for remote management of chronic diseases by presenting and evaluating an embedded and scalable distributed system using wearable sensors for the connection of cheap health devices based on prototyping eHealth platforms. The results of their analysis showed that portable devices (p << 0.01) are suitable for supporting the transmission and analysis of biometric signals into scalable telemedicine systems. In an observational study, Thakur et al. [5] presented a supervised ML-based model for predicting the clinical events during dialysis sessions using data from a non-contact sensor device. The authors found the findings and performance of the ML model quite encouraging and suggested the use of non-contact sensors in clinical settings for monitoring patients’ vital parameters and in early warning solutions for predicting the clinical events. In a study involving patients that recently had knee replacement surgery, Argent et al. [6] explored and evaluated the feasibility, usability, impact and user experience of an exemplar exercise biofeedback system for orthopedic rehabilitation at home. In order to maximize the engagement and impact, the study incorporated user-centered design approaches by incorporating participants’ evaluation during the design of the system. The findings of the study support the ongoing development and evaluation of sensor-based biofeedback systems, and authors found the system highly usable and effective for patient support and engagement. In a community health study, Martinez et al. [7] developed a new unsupervised exploratory method for characterizing feature extraction and detecting movement similarity in sleep by using actigraphy signals. The results of statistical analysis showed the potentials of this method for sleep disorders and their link with other conditions. The authors suggested the possible application of proposed approach for the extraction and comparison of sleep movements’ patterns in the field of medicine. Based on a previous work of a Wearable Heat stroke Detection Device (WHDD) [8] that was used for heat stroke prediction capability for any activity or exercise. Lin et al. [9] investigated the detailed information analysis and performed static and dynamic experiments for verifying the availability and effectiveness of WHDD experimental subjects. The results of their work demonstrated the superior applicability of the WHDD for predicting the occurrence of heat stroke effectively and ensuring the safety of runners. Using recurrent neural networks (RNNs)-based deep learning models, Luna-Perejon [10] presented a feasibility study of implementing a wearable system for the detection of falls and its associated risks/hazards in real time through accelerometer signals. Based on the results of the study, the authors recommended RNNs models as an effective method for creation of autonomous wearable fall detection systems in real time. Using a large real-world database of posture data, Stollenwerk et al. [11] analyzed the postural changes that are induced under postural training in three different positions, sitting, standing, and hip hinging, and compared the snapshots of unguided-guided posture pair based on features resulted from 2D spine curve geometry. The results showed the novelty of the work in the field of wearable-sensor-based evaluation of spine curves. Vega-Barbas et al. [12] proposed a precise and pervasive ergonomic platform for accurate assessment of continuous risk and personalized automated coaching by utilizing in-house developed garments and a mobile application. The results of the study demonstrating a good usability score proved the acceptable usability of the platform. The authors expected that wearable technology in the field of ergonomics can have cost effective risk assessment and economical solutions in the future. The study from Lin et al. [13] presented the design of a wearable cardiac health monitoring platform, implemented it as wearable smart clothing system with multi-channel mechanocardiograms and electrocardiograms measurements, and evaluated the usability of the system using technology acceptance model. The analysis and the results of the study showed the positive attitude of subjects for using this wearable system in providing early risk warnings. Based on deep learning, Lim et al. [14] presented a coaching assistant method to provide useful information for table tennis practice, and used long short-term memory (LSTM) recurrent neural networks (RNNs) with deep state space model and probabilistic inference to support practice. The promising results provided by this method showed its capability in characterizing high-dimensional time series patterns and providing useful information with wearable sensors in table tennis coaching. Lu et al. [15] developed and tested a new method that combined information from heart rate, respiration, and accelerations measurements to estimate energy expenditure. These data measurements were taken from wearable sensor system and were integrated by neural network based model. The results of the proposed method showed improved accuracy over two existing established methods. The authors suggested that this model along with wearable system could be utilized in both occupational as well as general health applications. Ejupi [16] investigated the feasibility of wearable textile-based sensors to accurately monitor breathing patterns, develop algorithm to detect talking using ML algorithm, and evaluate the model’s performance with the study participants. The evaluation showed random forest classifier as the best performer in the dataset. The authors suggested that this approach could be used to quantify talking through social interaction and prevent social isolation and loneliness. Using a previously developed inertial measurement unit device based on three sensor [17], Cesareo et al. [18] presented an automatic and position-independent algorithm to derive the respiration-induced movement and determine the respiratory rate accurately. The results showed that principal component analysis (PCA) fusion method obtained overall highest performance in terms of breathing frequency estimation, in both supine as well as seated position. The authors suggested that PAC fusion, as dimension-reduction method, can be used to analyze further data in the future. Using wearable technology and ML algorithms, Manjarres et al. [19] developed a smart physical workload tracking system in real time for simultaneous remote monitoring of people. The established framework was based on the concept of ergonomics to facilitate the work of health professionals and fitness experts. The results of two case studies in real time showed good accuracy and reliability of the system. The authors recommended the future developments by combining ergonomics and ML to predict the physical effort of activities and for injury prevention environments. Nam et al. [20] used an inertial measurement unit-based motion capture and analysis system to access arm movements. The study provided an important database on the dimensions of workspace and range of motions for arm movements. The validation results showed high accuracy and reliability of the system and emphasized on the importance of designing new exoskeletons for neurorehabilitation purposes. Zhang et al. [21] examined the relevance of different conventional physical activity metrics and complexity in the assessment of functional change after exercise intervention in younger and older adults. The findings of the study demonstrated the potential and usefulness of physical activity complexity metrics as compared to conventional metrics in assessment of functional changes for younger and older adults, and recommended them for the feasibility and effectiveness of risk identification and interventions. Hsu et al. [22] proposed a wearable 12-lead electrocardiogram monitoring system to measure the electrocardiogram (ECG) signals of patients with myocardial ischemia and arrhythmia. The experimental results of the study provided a good ECG signal quality even while walking and detected ECG features of the mentioned patients. The authors suggested the possible usefulness of the proposed system in future mobile ECG monitoring applications. Jayasinghe et al. [23] investigated and quantified the data received from sensors in different types of clothing in order to characterize the activities as compared to the body worn sensors’ data. The case study analysis indicated that clothing sensors data correlated well with the body worn sensors data, and classification results from clothing sensors were also promising compared to body-worn sensors. The results of the study showed potentials of this approach in daily monitoring. Allahbakhshi et al. [24] examined the role of Global Positioning System (GPS) sensors data for detection of physical activity in semi-structured and real-life protocols using participants with wearable devices in a study. The results provided insights in assisting physical activity for future study designs and guidance related to detection of posture and transport related motion activities. Cheung et al. [25] proposed a novel quantile coarsening analysis (QCA) for reducing the dimension of data from wearable devices and demonstrated the feasibility of this approach in a small cohort of relatively healthy individuals. Because of the versatility of the QCA approach, the authors suggested that it can provide useful analytical tools for data in multi-modal monitoring. By explaining the role of actigraphs in personalized health, fitness monitoring and Internet of Medical Things (IoMT) paradigm, Athavale [26] presented a study utilizing wearable devices to capture and analyze physiological data at home-based health monitoring in an IoMT environment, and proposed a low level encoding scheme to improve actigraphy analysis. In order to ensure that there was no loss of information in encoding process, ML approach was used for the study validation. Based on the dataset Personal RIsk DEtection (PRIDE) [27], a study by Trejo [28] first explored the impact of using dimension reduction techniques and frequency domain features for personal risk detection through correlation matrix and principal component analysis, and then efficiently accelerated the training and classification process of a given classifier for mobile devices. The results of the study were encouraging for timely detection of risk prone situations that can threaten a person’s physical integrity. Yurtman et al. [29] proposed a methodology to transform the recorded motion sensor sequences to sensor unit orientation unchangeably and incorporated it in pre-processing stage of the standard activity recognition scheme. The results from comparative evaluation of proposed method with the existing state-of-the-art classifiers showed its substantially better output in classifying stationary activities and hence its possible application in various wearable systems. Dutta et al. [30] used a novel framework to classify and model the physical activities performed by different participants in a supervised lab-based protocol and then utilized it to identify the physical activities in a free-living setting using the data from wrist worn accelerometers. The positive results of the study demonstrated its application for estimating physical activities in future cohort or intervention studies. In a study, Rosati et al. [31] compared two different feature sets for real-time human activity recognition (HAR) applications; one comprising time, frequency, and time-frequency related parameters used in the literature and the other containing only time-related variables linked with biomechanical meaning of acquired signals. The results showed that both set of features can reach high accuracy with support vector machine (SVM) classifier, but the new proposed variables can be easily interpreted and employed for better understanding of the alterations of biomechanical behavior in complex situations. In a study focusing on healthy subjects having normal heart activity, Morelli et al. [32] investigated the effects of interpolation on time and duration with increasing missing values to assess the interpolation strategy for better results during the estimation of heart rate variability (HRV) features. The results concluded that interpolation in time is the most favorable method for producing better HRV features estimation as compared to interpolation on duration. Fortin-Cote et al. [33] presented a graphical software for the visualization and preprocessing of raw data received from accelerometer for human posture tracking and assessment. This tool was aimed to provide support for calibration of orientation estimate of inertial measurement units (IMUs) that are used for joint angle measurement. Two case studies were used to demonstrate the usefulness of this open source software. Broadley et al. [34] presented a systematic review to assess existing methods of evaluating fall detection systems, identify their limitations, and propose improved evaluation methods in the literature. The search results of articles that met the inclusion criteria identified few issues, such as use of small population datasets and inconsistency for performance quantification for these systems. Sensitivity, precision, and F-measures were derived as the most appropriate and robust measures for their realistic performance evaluation. 
